# Targeting WHO-Priority MDR and XDR Gram-Negative Ocular Isolates by Combining Conventional Antimicrobials with Membrane-Active Peptides

**DOI:** 10.3390/antibiotics15080793

**Published:** 2026-08-16

**Authors:** Teshome Belachew Eshete, Shyam Kumar Mishra, Naresh Kumar, Mark Willcox

**Affiliations:** 1 School of Optometry and Vision Science, Faculty of Medicine and Health, University of New South Wales, Sydney, NSW 2052, Australia; s.mishrabaishnab@unsw.edu.au; 2 Department of Microbiology, Maharajgunj Medical Campus, Institute of Medicine, Tribhuvan University, Kathmandu 44600, Nepal; 3 School of Chemistry, Faculty of Science, University of New South Wales, Sydney, NSW 2052, Australia; n.kumar@unsw.edu.au

**Keywords:** ocular pathogens, multidrug resistance, antimicrobial peptides, synergistic combinations, colistin, Mel4

## Abstract

**Background/objectives**: Multidrug-resistant and extensively drug-resistant Gram-negative pathogens are a major cause of severe ocular infections, yet treatment options are increasingly compromised by escalating antibiotic resistance. Membrane-active peptides (colistin and Mel4) offer the potential to restore antibiotic susceptibility. This study investigated the efficacy of peptide- and colistin-based adjuvant strategies in enhancing the activity of conventional antibiotics. **Methods**: Antibacterial activity was assessed using MIC/MBC testing, checkerboard assays, time–kill kinetics, and biofilm disruption studies, supported by confocal microscopy. Toxicity was assessed using L929 fibroblasts and red blood cells. A mechanistic study was performed with a membrane permeability assay. **Results**: Clinical isolates of *Pseudomonas aeruginosa*, *Acinetobacter baumannii*, and *Klebsiella pneumoniae* showed high resistance to six antibiotic classes (up to MICs > 2048 mg/L), while polymyxin B and colistin remained active (MIC ≤ 2 mg/L). Mel4 displayed variable activity (MIC 7.8–500 mg/L). A total of 102 antibiotic–antibiotic, antibiotic–Mel4, and antibiotic–colistin combination tests were performed, of which 45 showed synergistic interactions. Combining traditional antimicrobials with either colistin or Mel4 restored antibiotic susceptibility, reducing MICs up to 2048-fold. Aminoglycoside–colistin combinations significantly lowered MICs, especially against *K. pneumoniae*. Time–kill assays confirmed rapid bactericidal effects (>3 log10 reduction in 3 h). The ciprofloxacin–Mel4 combination effectively disrupted biofilms (62–92%) with low toxicity and high cell viability. Membrane permeability assays showed that ciprofloxacin has limited activity, whereas both Mel4 and the ciprofloxacin–Mel4 combination showed enhanced activity across concentration gradients and over time. **Conclusions**: Membrane-active antimicrobials, colistin and Mel4, enhance conventional antibiotics against multidrug- and extensively drug-resistant ocular Gram-negative pathogens by restoring susceptibility, accelerating bactericidal effects, and disrupting biofilms. With low toxicity, these combinations represent promising therapeutic strategies for severe multidrug-resistant ocular infections.

## 1. Introduction

Ophthalmic infections can cause visual impairment and irreversible blindness if not promptly treated [1]. Several ESKAPEE pathogens, including *Pseudomonas aeruginosa*, *Acinetobacter baumannii*, *Klebsiella pneumoniae*, and *Escherichia coli*, are associated with ocular infections and are increasingly difficult to treat because of antimicrobial resistance [2,3,4,5,6,7]. *P. aeruginosa* is a leading cause of bacterial keratitis, particularly in contact lens-associated infections [4] and increasing resistance to antibiotics has been associated with poorer clinical outcomes [8,9]. Biofilm formation further complicates treatment by markedly reducing antimicrobial susceptibility [10].

Combination antibiotic therapy is commonly used for severe bacterial keratitis to broaden antimicrobial coverage and improve treatment efficacy [11,12,13]. However, conventional antibiotic combinations may still select for resistant subpopulations [14]. Combining antibiotics with membrane-active agents, such as colistin, polymyxin B, or antimicrobial peptides (e.g., Mel4), may enhance bacterial membrane permeability, restore antibiotic susceptibility, and reduce the concentrations required for antimicrobial activity [15,16,17,18,19].

Mel4 is a short, 17-amino-acid peptide with the sequence KNKRKRRRRRRGGRRRR, and a predicted net charge of +14 at neutral pH [20,21]. Unlike many membrane-active antimicrobial peptides, Mel4 contains no hydrophobic amino acid residues and has a very low predicted hydrophobic moment of 0.039 [20]. Despite this low hydrophobicity, Mel4 can interact with bacterial envelope components, neutralize lipopolysaccharide, rapidly depolarize the membrane of *P. aeruginosa*, and induce ATP leakage, membrane permeabilization, and loss of intracellular contents [20].

When covalently immobilized on contact lenses, Mel4 retained antimicrobial activity without substantially altering clinically relevant lens parameters. Mel4-coated lenses showed no evidence of ocular irritation in rabbit studies and were well tolerated in human contact-lens trials [22,23,24]. In an extended-wear Phase 3 clinical study involving 176 participants, no significant differences were observed between Mel4-coated and control lenses in ocular redness, corneal staining, dryness, or subjective comfort [24]. These preclinical and clinical ophthalmic evaluations make Mel4 particularly relevant to the present study, which investigates its ability to enhance the activity of different classes of antibiotics against multidrug-resistant (MDR) and extensively drug-resistant (XDR) ocular clinical isolates [20].

However, the development of these combination therapies remains challenging due to factors such as unpredictable interactions, leading to toxicity [25]. Therefore, advances in predicting toxicological outcomes and understanding drug interaction profiles are crucial to ensure the safety and efficacy of such approaches [26]. The present study evaluated the synergistic effects between conventional antibiotics and membrane-active antimicrobials against multidrug-resistant ESKAPEE strains and assessed the safety of the combinations.

## 2. Results

### 2.1. Antimicrobial Resistance Patterns of MDR/XDR Gram-Negative Pathogens

All isolates exhibited variable resistance profiles against conventional antibiotics and the membrane-active antimicrobials. Notably, *A. baumannii*, *K. pneumoniae*, and some *P. aeruginosa* strains showed high levels of resistance to six tested conventional antimicrobials (ciprofloxacin (CIP), ceftazidime (CAZ), levofloxacin (LEV), gentamicin (GEN), tobramycin (TOB), and meropenem (MER), ranging from 8 to >2048 mg/L (Table 1).

Almost all tested Gram-negative pathogen strains were intermediate to polymyxin B (PMB) and colistin (COL), with MIC values of <2 mg/L (Table 2).

### 2.2. In Vitro Evaluation of Synergy by Checkerboard Assay

The effectiveness of different combinations of aminoglycosides with cephalosporin is presented in Table 3. Overall, whilst synergy was observed for five strains and additive action for the remainder, the combinations often failed to reduce the MICs of the individual antibiotics below the resistant levels. For example, the combination of gentamicin and ceftazidime was synergistic with *P. aeruginosa* 31, but MICs remained above the susceptibility threshold (≤0.5 mg/L and ≤2-8 mg/L, respectively). As this may still pose a challenge to therapeutic success, combinations of traditional antibiotics and membrane-active compounds were investigated.

The synergy between traditional antibiotics and colistin against drug-resistant ESKAPEE pathogens is presented in Table 4. Overall, the combinations produced synergistic, additive, or indifferent interactions, with no antagonistic effects observed. Whilst synergy occurred, this did not mean that the MIC of the traditional antibiotic was always reduced to or below the susceptibility cut-off point. Unlike Mel4–antibiotic combinations (Table 5), which showed limited activity against *K. pneumoniae*, 8 of the 13 (61.5%) colistin-containing combinations exhibited additive or synergistic interactions. Combining colistin with gentamicin, ceftazidime, meropenem, or tobramycin substantially reduced antibiotic MICs, with reductions of up to 2048-fold observed in selected *K. pneumoniae* isolates.

Combinations of Mel4 with conventional antimicrobials demonstrated synergistic or Indifferent effects against *P. aeruginosa* and *A. baumannii*, with MIC reductions of up to 256-fold and 4096-fold, respectively, while *K. pneumoniae* showed a more limited response (<8-fold decrease) (Table 5).

**Figure 1:**
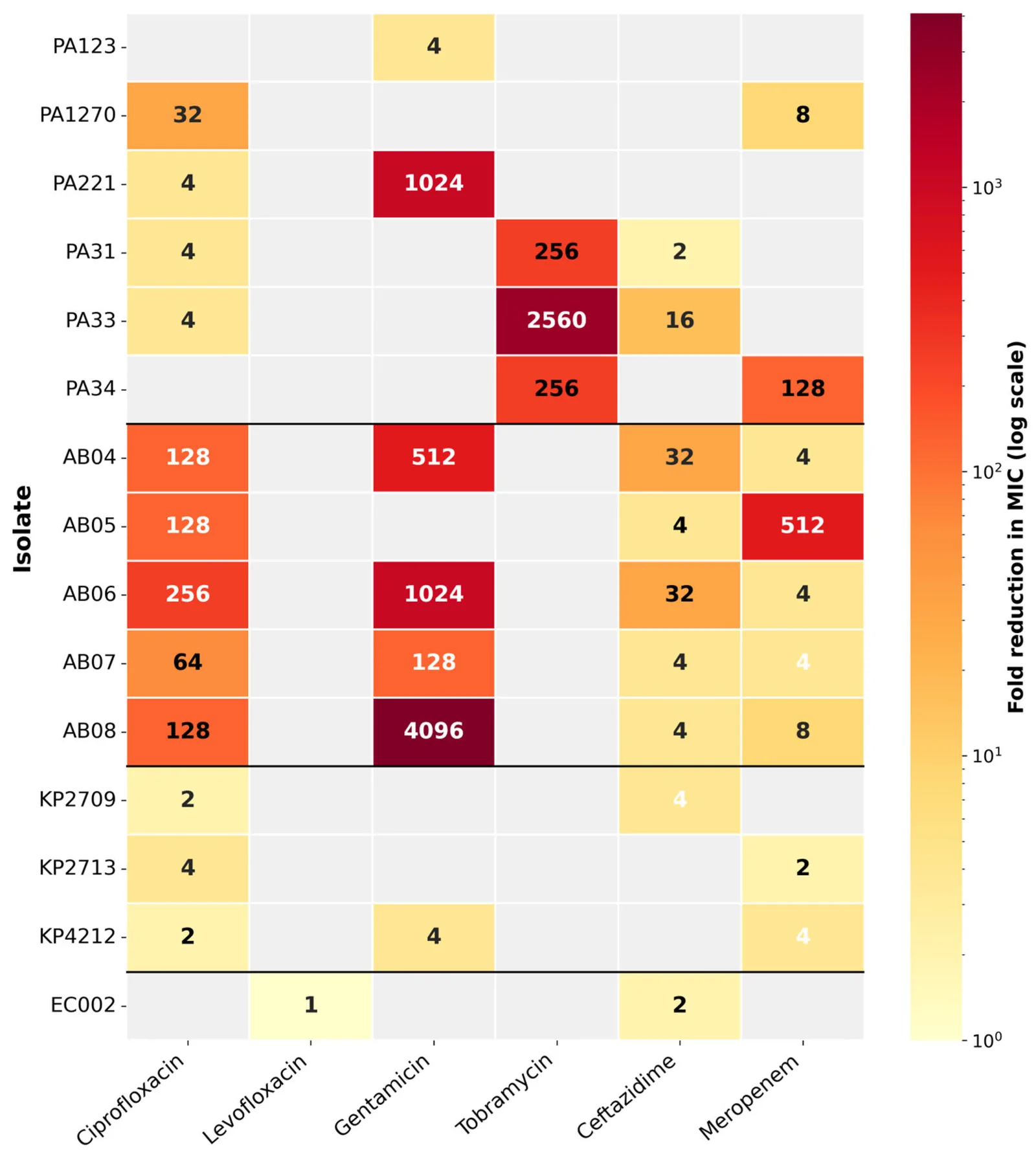
Fold reduction in antibiotic MICs when combined with Mel4. Heatmap showing the fold reduction in the MIC of each antibiotic when tested in combination with Mel4 against the selected bacterial isolates. Fold reduction was calculated as the MIC of the antibiotic alone divided by its MIC in combination with Mel4. The numerical values within the cells represent the fold reduction, while increasingly darker colors indicate greater MIC reductions on a logarithmic scale. Gray cells indicate combinations that were not tested or for which data were unavailable. Horizontal black lines separate *P aeruginosa* (PA), *A. baumannii* (AB), *K. pneumoniae* (KP), and *E. cloacae* (EC) isolates.

The fold reduction in tested antibiotics-Mel4 combinations is provided in the heatmap (Figure 1).

### 2.3. Time–Kill Assay Results

Representative ocular isolates showing synergism in the antibiotic–Mel4 checkerboard assay were selected for time–kill analysis. The meropenem–Mel4 combination was synergistic against *A. baumannii* strain 08 at 64 mg/L meropenem and 62.5 mg/L Mel4. Similarly, ciprofloxacin–Mel4 demonstrated synergy against *P. aeruginosa* strains 31 and 33 at 4 mg/L ciprofloxacin and 15.625 mg/L Mel4. Viable bacterial counts were determined at 0, 3, 6, 9, 12, and 24 h.

The time–kill curves demonstrated that Mel4 markedly enhanced the bactericidal activity of both antibiotics, whereas the individual agents produced little or only transient killing. Against *A. baumannii* strain 08, Mel4 alone caused minimal inhibition, while meropenem alone produced an initial reduction of approximately 2 log_10_ CFU/mL at 3 h, followed by rapid regrowth to about 7.5 log_10_ CFU/mL. In contrast, the meropenem–Mel4 combination reduced bacterial counts below the plotted detection limit by 3 h and maintained this effect through 12 h. Although regrowth occurred by 24 h, the combination remained approximately 4 log_10_ CFU/mL below either monotherapy, meeting the accepted definition of synergy, namely a reduction of at least 2 log_10_ CFU/mL relative to the most active single agent.

**Figure 2:**
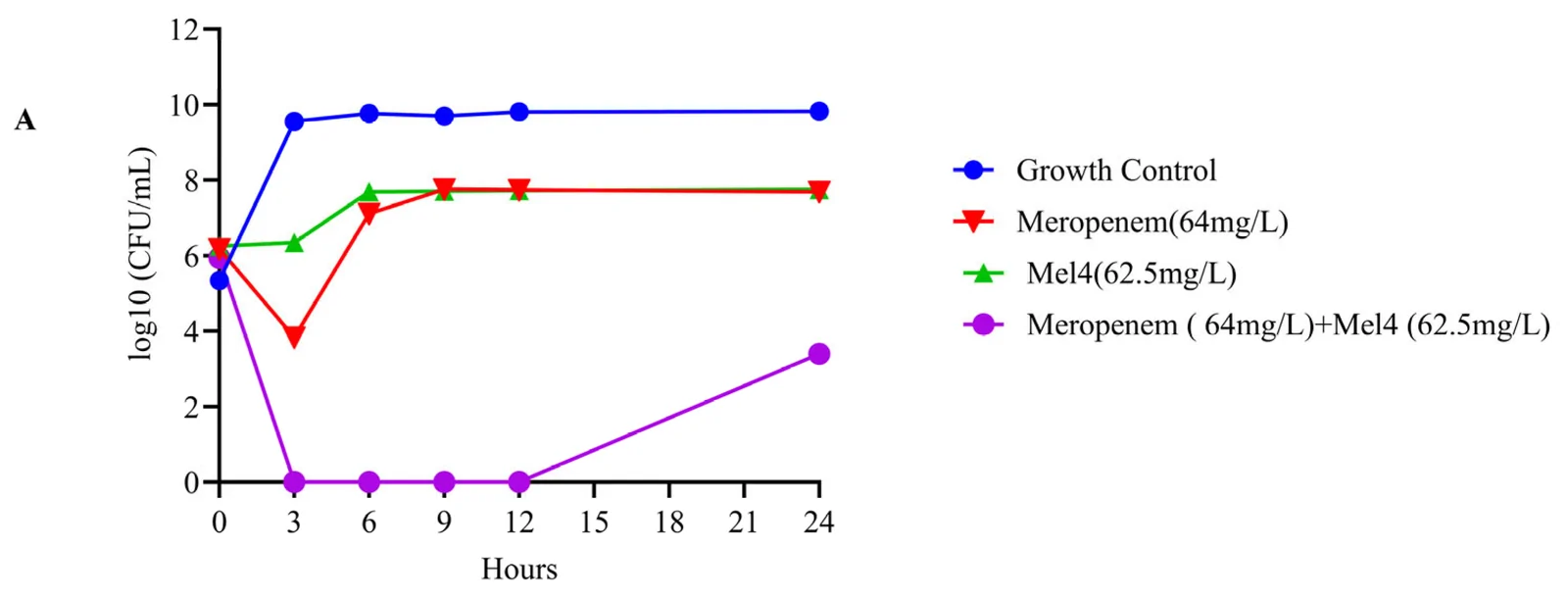

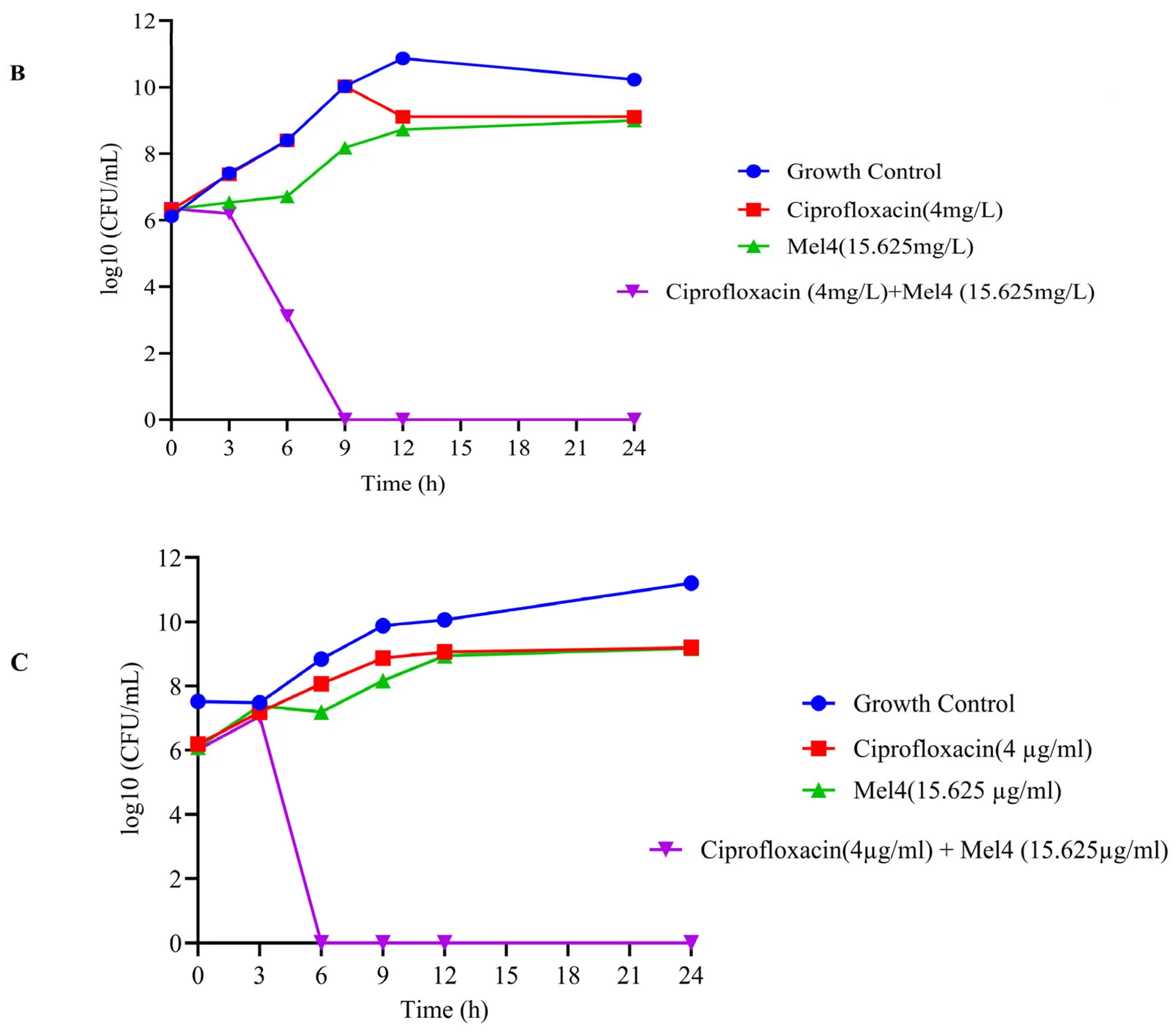
Time–kill assay of individual antibiotics and combinations against *A. baumanii* 08 (**A**), *P. aeruginosa* 31 (**B**), and *P. aeruginosa* 33 (**C**).

Against *P. aeruginosa* strains 31 and 33, ciprofloxacin and Mel4 alone did not produce meaningful killing, and bacterial counts increased to approximately 9 log_10_ CFU/mL by 24 h. The combination, however, produced a progressive decline from approximately 6 log_10_ CFU/mL at baseline to around 3 log_10_ CFU/mL by 6 h. Counts fell below the detection limit by 9 h for strain 31 and by 6 h for strain 33, remaining undetectable through 24 h. These findings confirm rapid, sustained bactericidal synergy between ciprofloxacin and Mel4. No comparable reduction was observed with either monotherapy, highlighting the potentiating effect of Mel4 on antibiotic activity against resistant isolates (Figure 2).

### 2.4. Biofilm Disruption Assay Results

**Figure 3:**
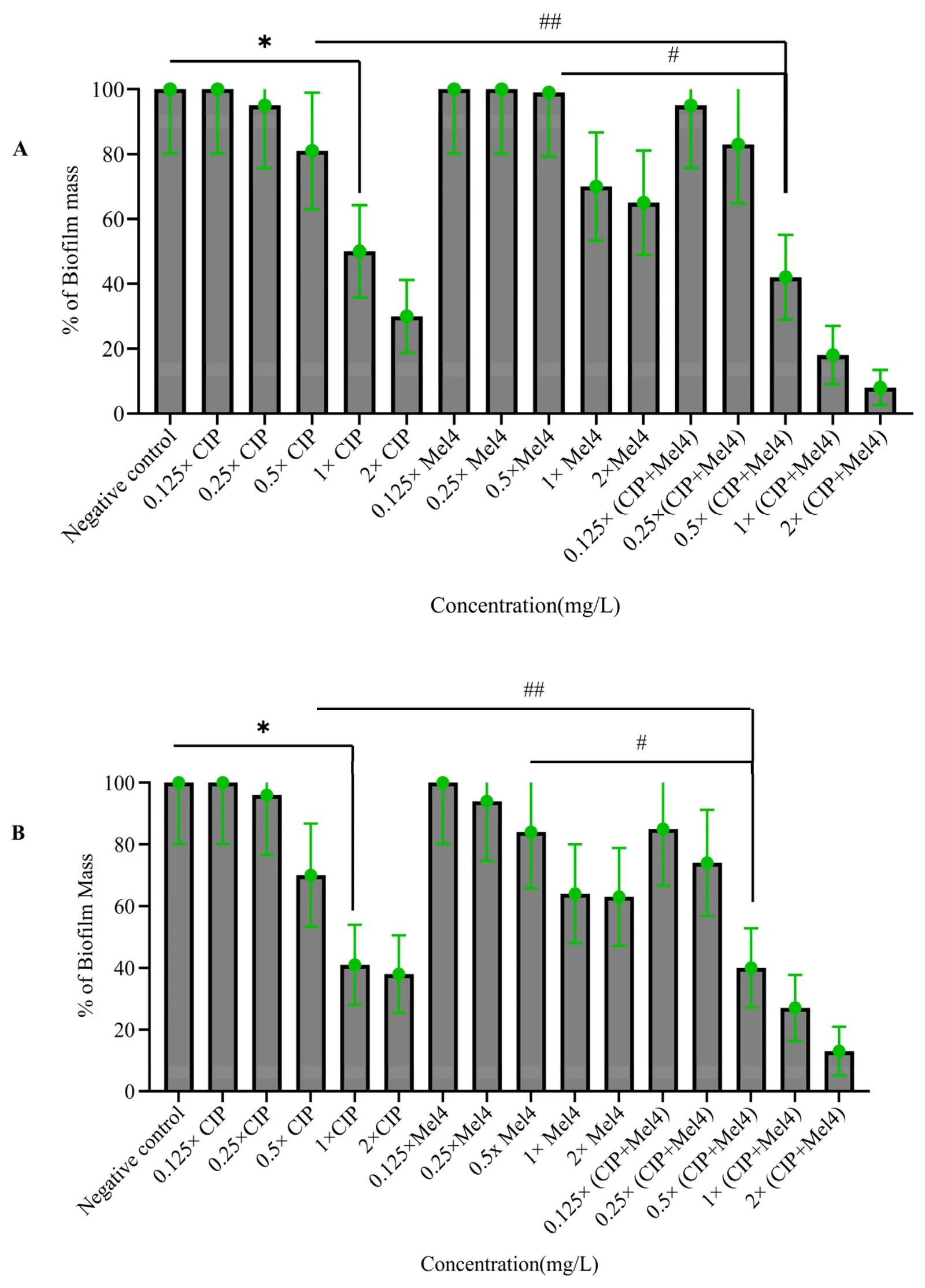
Biofilm degradation by ciprofloxacin, Mel4, and their combination against *P. aeruginosa* 31. Preformed biofilms of MDR *P. aeruginosa* strains 31 (**A**) and 33 (**B**) were disrupted by various concentrations of ciprofloxacin, Mel4, and their combination. * Represents a significant (*p* < 0.001) decrease compared to the negative control (bacteria grown in the absence of antibiotics). # indicates significant (*p* < 0.001) decrease for the combinations compared to Mel4 alone. ## indicates a significant (*p* < 0.001) decrease for the combinations compared to ciprofloxacin alone. Negative control = bacteria grown in the absence of antimicrobials; CIP = ciprofloxacin.

The ability of ciprofloxacin, Mel4, and their combination at different concentrations, including their sub-MIC levels, to degrade preformed biofilms was evaluated against *P. aeruginosa* strains 31 and 33. Ciprofloxacin showed minimal activity at 1/8×, 1/4×, and 1/2× MIC levels against both strains, with less than 25% biofilm reduction (Figure 3). However, as the concentration increased, biofilm reduction increased, reaching 59-70% at 1× and 2× MIC levels (*p* < 0.001). In contrast, Mel4 showed relatively small reductions in biofilm mass at 1× and 2× MIC, with reductions of 35–37%. However, the ciprofloxacin–Mel4 combination demonstrated a synergistic effect, achieving biofilm degradations of 62–92% at 1/2×, 1×, and 2× MIC levels (*p* < 0.001).

### 2.5. Confocal Microscopy Results

**Figure 4:**
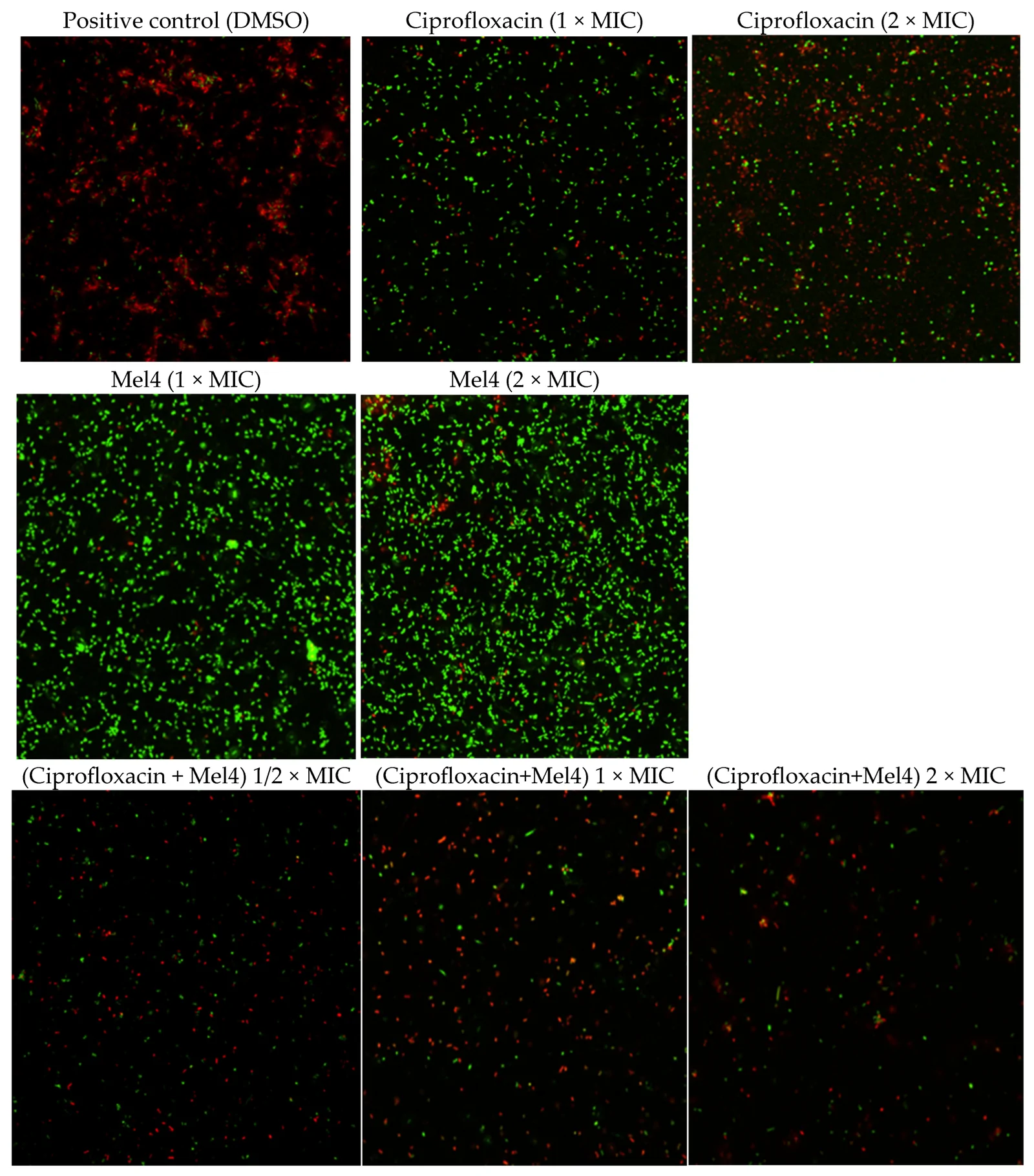
Effects of ciprofloxacin and Mel4 monotherapy and combination treatments on *P. aeruginosa* strain 31 biofilm viability. A positive control treated with 100% DMSO stained predominantly red, indicating non-viable cells. Treatment of the biofilms with ciprofloxacin at 1× MIC and 2× MIC resulted in a substantial number of red-stained cells, indicating bacterial cell death. In contrast, biofilms treated with Mel4 alone at 1× MIC and 2× MIC exhibited minimal reduction in biofilm biomass, as evidenced by the predominance of green, fluorescent cells, indicating a high proportion of viable bacteria within the biofilm structure. However, treatment with the combination of ciprofloxacin and Mel4 resulted in enhanced bactericidal activity against the biofilm at 1/2× MIC, 1× MIC, and 2× MIC.

During the crystal violet staining assay, both *P. aeruginosa* strain 31 and strain 33 showed similar responses to biofilm degradation following treatment with ciprofloxacin, Mel4, and their combinations. Therefore, subsequent confocal laser scanning microscopy (CLSM) analysis was performed using *P. aeruginosa* strain 31 only (Figure 4).

### 2.6. Preliminary Cytotoxic Effects of Ciprofloxacin, Meropenem, and Mel4

**Figure 5:**
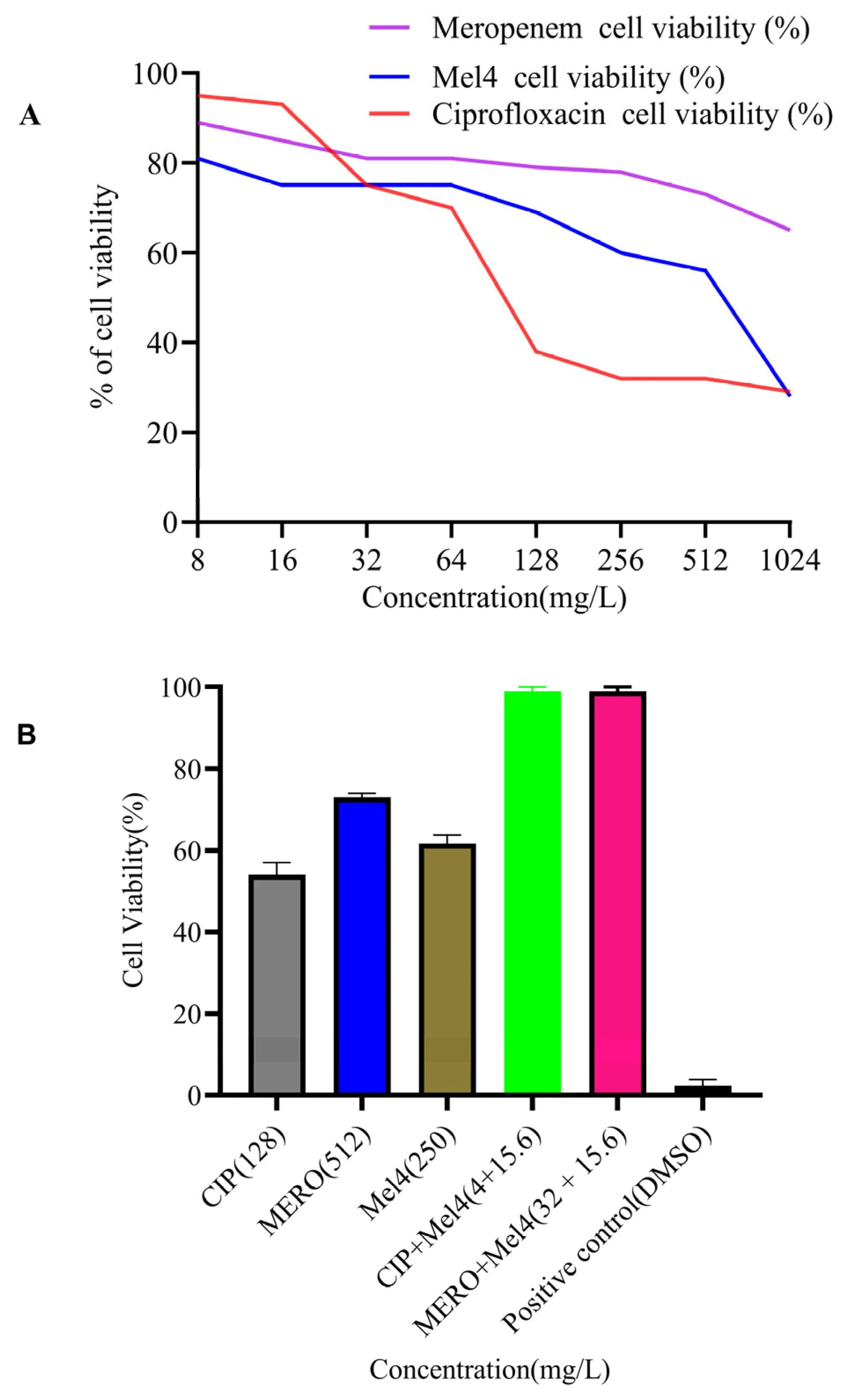
A preliminary assessment of the cytotoxicity of the antimicrobials on L929 cells. (**A**) Cell viability over a concentration range for the individual antimicrobials; (**B**) cell viability of each antimicrobial alone at its MIC or in combination at the lowest concentration that produced synergy, compared to the positive control (DMSO).

To evaluate cytotoxicity, ciprofloxacin, meropenem, and Mel4 were tested individually and in combination using MTT assays on L-929 mammalian cells, along with hemolysis assays. Across concentration gradients, all antimicrobials maintained >70% cell viability up to 64 mg/L, although toxicity increased at higher concentrations. The IC_50_ values indicated varying toxicity, with ciprofloxacin (93.46 mg/L) being more toxic than Mel4 (440.2 mg/L) and meropenem (1434 mg/L). At their MICs, ciprofloxacin reduced cell viability to 54%, whereas Mel4 and meropenem maintained 60% and 73% viability, respectively. Notably, ciprofloxacin–Mel4 and meropenem–Mel4 combinations at synergistic concentrations showed no cytotoxicity (Figure 5).

### 2.7. Preliminary Hemolysis Effects of Ciprofloxacin and Mel4

Mel4 demonstrated no hemolysis across all tested concentrations (32–512 mg/L). In contrast, ciprofloxacin exhibited concentration-dependent hemolysis, reaching 39% at 512 µg/mL. Notably, ciprofloxacin–Mel4 combinations demonstrated minimal hemolysis (0–1%) across all concentrations, including up to 3× MIC, indicating a safety profile with reduced cytotoxicity (Table 6).

### 2.8. Mechanistic Studies

#### Cell Membrane Depolarization

**Figure 6:**
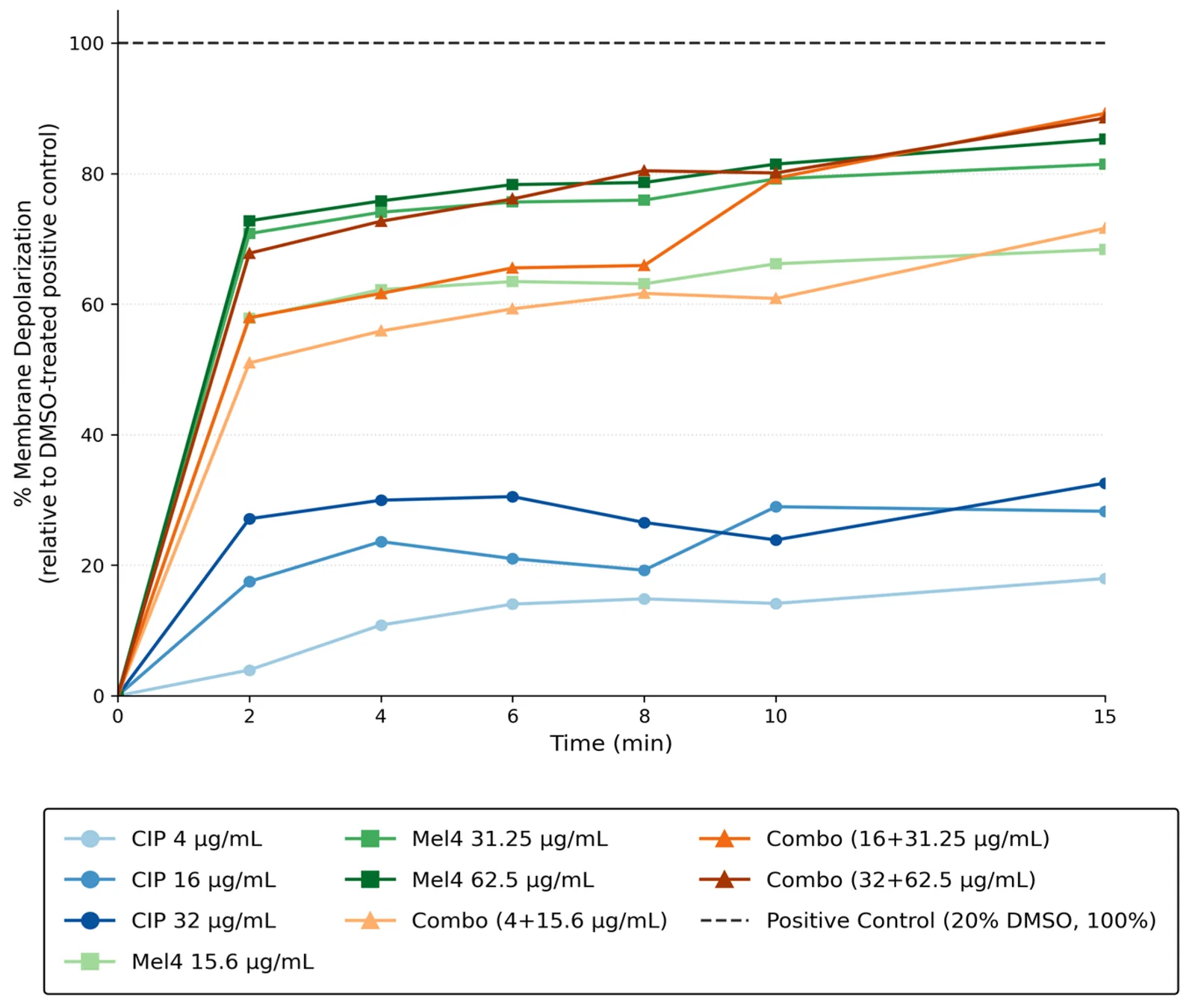
Time-dependent membrane depolarization of bacterial cells following treatment with ciprofloxacin (CIP; 4, 16, and 32 mg/L), Mel4 (15.6, 31.25, and 62.5 mg/L), and combinations of CIP and Mel4 over a 15 min period. Membrane depolarization was measured as a percentage relative to the DMSO-treated positive control (100%).

Membrane depolarization assay was performed against *P. aeruginosa* over 15 min following exposure to ciprofloxacin, Mel4, and their combinations at a synergistic concentration (4 mg/L of ciprofloxacin, 15.6 mg/L of Mel4), 1× MIC (16 mg/L of ciprofloxacin and 31.5 mg/L of Mel4 and 2× MIC (32 mg/L of ciprofloxacin and 62.6 mg/L of Mel4). Membrane depolarization occurred rapidly following exposure to Mel4 and Mel4–ciprofloxacin combinations, with substantial increases observed within the first 2 min. Mel4 alone induced concentration-dependent membrane depolarization, reaching approximately 83–86% at 15 min. In contrast, ciprofloxacin alone caused only modest depolarization (18–32%), indicating minimal direct effects on bacterial membrane potential. Combination treatments exhibited markedly greater depolarization than ciprofloxacin alone, reaching 71–89% by 15 min. The highest response was observed with the combination of 16 mg/L ciprofloxacin and 31.25 mg/mL Mel4 (approximately 89%), suggesting that Mel4-mediated membrane disruption may facilitate ciprofloxacin’s antibacterial activity by promoting its intracellular access (Figure 6). A ciprofloxacin intake assay may further confirm this preliminary result.

## 3. Discussion

In this study, the antimicrobial activities of traditional antibiotics, the novel membrane-active peptide Mel4, and polymyxin (colistin), either individually or in combination, were evaluated against both planktonic and sessile cells of selected ESKAPEE pathogens. Additionally, the preliminary cytotoxic and hemolytic effects of these compounds were examined individually and in combination at sub-MIC concentrations.

Although antibiotics have made remarkable contributions to human and animal health, their indiscriminate and prolonged use has accelerated the emergence of multidrug-resistant (MDR) bacteria, rendering many standard monotherapies increasingly ineffective and difficult to manage [16]. Consistent with this growing clinical challenge, the isolates examined in the present study exhibited high levels of antimicrobial resistance. Notably, *P. aeruginosa*, *A. baumannii*, and *K. pneumoniae* demonstrated substantial resistance to β-lactams (ceftazidime, meropenem), fluoroquinolones (ciprofloxacin, levofloxacin), and aminoglycosides (gentamicin), with minimum inhibitory concentrations (MICs) exceeding 2048 mg/L.

In contrast, polymyxin B and colistin retained potent antibacterial activity, with MIC values generally ranging from 0.5 to 2 mg/L. However, these agents are typically reserved as last-line treatments because of concerns regarding toxicity and the potential emergence of polymyxin resistance. Their retained activity therefore does not eliminate the need for safer and more effective therapeutic strategies against MDR pathogens.

Combination therapy may restore antibiotic activity against resistant isolates by reducing MICs, enhancing bacterial killing, and limiting exposure to suboptimal drug concentrations that promote further resistance [28,29,30,31]. Consistent with this, the combination of aminoglycosides with colistin demonstrated pronounced synergistic or additive effects, with gentamicin–colistin and tobramycin–colistin combinations reducing aminoglycoside concentrations by up to 32-fold against *A. baumannii* and up to 256-fold against *K. pneumoniae*.

Similarly, cephalosporin (ceftazidime–colistin) combinations showed substantial efficacy in the present study. Notably, 62.5% (5/8) of ceftazidime–colistin combinations restored ceftazidime susceptibility, reducing the concentrations required for resistance to below the CLSI susceptibility breakpoint (<8 mg/L). [32]. Furthermore, combinations of colistin with ciprofloxacin or meropenem exhibited the most pronounced effects, with nearly all combinations reducing resistant concentrations of ciprofloxacin and meropenem to susceptible levels, achieving up to a 2048-fold reduction. Collectively, these results underscore the potent synergistic potential of colistin-based combination therapies in restoring antibiotic susceptibility against MDR Gram-negative pathogens.

The activity of Mel4 in combination with conventional antibiotics was also examined. Previous investigations of Mel4 have largely focused on its activity as a free peptide or contact-lens coating, its membrane-active mechanism, and its ability to inhibit or disrupt *P. aeruginosa* biofilms [22,23,33]. Mel4 has been shown to interact with lipopolysaccharide and rapidly depolarize and permeabilize the *P. aeruginosa* cytoplasmic membrane. An earlier pilot checkerboard study involving a limited number of *P. aeruginosa* and *Staphylococcus aureus* strains investigated the Mel4–ciprofloxacin combination, although only an additive result with FICI of 0.56 was reported against t *P. aeruginosa* [21]. This suggests that the interaction may depend on the bacterial strain, resistance phenotype, concentration ratio, and experimental conditions.

In contrast, the present study identified synergistic or additive Mel4 combinations among highly resistant clinical isolates. Mel4 enhanced the activity of ciprofloxacin, meropenem, gentamicin, and tobramycin, producing MIC reductions of up to 256-fold against *P. aeruginosa* and up to 4096-fold against *A. baumannii*. Importantly, Mel4 combinations reduced ciprofloxacin and meropenem MICs below the CLSI susceptibility breakpoints against the XDR *P. aeruginosa* isolate PA1270. These findings extend the earlier pilot study by demonstrating that Mel4 can potentiate intracellularly acting antibiotics against MDR and XDR clinical isolates, including clinically relevant ocular isolates. The limited reductions observed against *K. pneumoniae* further indicate that Mel4 potentiation is species- and strain-dependent rather than a universal response among Gram-negative pathogens.

Biofilm formation represents a major obstacle in the management and eradication of bacterial infections. Conventional antibiotics and antimicrobial peptides (AMPs) that are effective against planktonic bacterial cells often fail to eliminate biofilm-associated bacteria, as biofilms confer significantly higher levels of antimicrobial tolerance compared to their planktonic counterpart [20]. Previous work also demonstrated that Mel4 and ciprofloxacin can enhance the disruption of established *P. aeruginosa* biofilms [20]. The present results are consistent with those observations but extend them to strong biofilm-producing clinical isolates and include evaluation at sub-MIC concentrations against two strong biofilm-producing *Pseudomonas aeruginosa* strains (31 and 33). Mel4 alone showed relatively limited antibiofilm activity, whereas its combination with ciprofloxacin produced greater reductions in biofilm biomass or viability than either individual treatment. This synergism may stem from the multifaceted mechanisms of antimicrobial peptides (AMPs), including their ability to cleave peptidoglycan, disrupt membrane integrity and potentially neutralize or disassemble lipopolysaccharides, inhibit cell division and survival, modulate adhesion molecule synthesis and function, and suppress the bacterial stringent response [34,35,36].

Selectivity toward prokaryotic cells is a crucial parameter when evaluating the potential clinical applicability of novel antimicrobial peptides (AMPs) and antibiotics, either as standalone agents or in combination with conventional antimicrobials [37]. In this study, the potential cytotoxic effects of Mel4 alone at its MIC and in combination with ciprofloxacin and meropenem at sub-MIC concentrations were evaluated using MTT and hemolysis assays on L929 mammalian fibroblast-like cells and horse red blood cells, respectively. Although L929 cells are recommended in both ISO 10993-5, Biological Evaluation of Medical Devices—Part 5: Tests for In Vitro Cytotoxicity and ISO 18189:2016, Ophthalmic Optics—Contact Lenses and Contact Lens Care Products—Cytotoxicity Testing of Contact Lenses in Combination with Lens Care Solution to Evaluate Lens/Solution Interactions [38,39], findings obtained using this non-ocular cell line still have limited relevance for assessing ocular safety. Further evaluation using ocular cell models and in vivo tolerance studies is required.

Mel4 exhibited cytotoxicity, reducing cell viability to 28% at 1000 mg/L, whereas approximately 60% viability was maintained at its MIC. Interestingly, cell viability markedly improved when Mel4 was combined with ciprofloxacin, meropenem, or erythromycin at sub-MIC levels (1/4 MIC), maintaining 100% viability. The calculated IC_50_ value of Mel4 was 440.2 mg/L. Given that the MIC value of Mel4 against *A. baumannii* was 250 mg/L, the resulting selectivity index (SI = IC_50_/MIC) was 1.76, indicating moderate selectivity toward bacterial cells. However, Mel4 exhibited only 1% hemolytic activity between 128 and 512 mg/L, with no detectable hemolysis at lower concentrations. The ciprofloxacin–Mel4 combination at sub-MIC concentrations also induced minimal hemolysis (1%) at five- and six-fold multiples of the combination sub-MIC, while no hemolytic effect was observed at other concentrations.

The membrane-depolarization findings provide further biological support for the observed interaction. Previous mechanistic studies established that Mel4 can rapidly disrupt the membrane potential of *P. aeruginosa*. In the present study, Mel4 and Mel4–ciprofloxacin combinations caused substantially greater membrane depolarization than ciprofloxacin alone, including at the synergistic concentrations of 4 mg/L ciprofloxacin and 15.6 mg/L Mel4. These results are consistent with the hypothesis that Mel4-mediated membrane disruption may increase bacterial susceptibility to ciprofloxacin. Nevertheless, membrane depolarization does not directly demonstrate increased intracellular ciprofloxacin accumulation. Therefore, the results should be interpreted as mechanistic support for, rather than definitive proof of, enhanced ciprofloxacin entry. A ciprofloxacin uptake assay will be required to confirm this proposed mechanism.

A key novelty of the present study is not simply the observation that membrane-active agents can enhance antibiotic activity. Rather, it is the systematic, side-by-side evaluation of colistin and Mel4 as antibiotic-potentiating agents against MDR and XDR Gram-negative pathogens. The study connects changes in planktonic susceptibility with time-dependent killing, activity against strong biofilm-producing isolates, membrane depolarization and preliminary mammalian cell and hemolysis assessments. This integrated approach provides a broader assessment of efficacy, potential mechanism and preliminary safety than previous studies that examined these aspects separately. It also identifies Mel4 as a potential non-polymyxin antibiotic adjuvant whose activity warrants further evaluation in corneal epithelial cells and experimental models of bacterial keratitis.

This research has strengths and weaknesses. The major strength of this study is its systematic evaluation of conventional antibiotics in combination with the membrane-active peptide Mel4 and polymyxins against multidrug-resistant Gram-negative bacteria, including clinically relevant ocular isolates. The antimicrobial interactions were assessed using complementary methods, including checkerboard, time–kill, and biofilm assays, which provided more robust evidence than reliance on a single susceptibility method. The inclusion of highly resistant isolates also allowed the study to evaluate whether combination therapy could restore or enhance the activity of antibiotics against difficult-to-treat pathogens. In addition, the assessment of cytotoxicity and hemolytic activity provided important preliminary safety information. The research may also be useful in the future when further outbreaks of keratitis are caused by highly antibiotic-resistant strains, as occurred recently with *P. aeruginosa* [38] that resulted in four deaths and four enucleations. These strains were resistant to polymyxins as well as cefepime, ceftazidime, piperacillin-tazobactam, aztreonam, carbapenems, ceftazidime-avibactam, ceftolozane-tazobactam, fluoroquinolones, amikacin, gentamicin, and tobramycin https://archive.cdc.gov/www_cdc_gov/hai/outbreaks/crpa-artificial-tears.html (accessed on 14 June 2026). Combinations of polymyxins with fluoroquinolones or aminoglycosides may have been useful. Overall, the study provides clinically relevant evidence supporting antibiotic–peptide combination therapy as a potential strategy for managing resistant ocular infections and identifies promising combinations for further investigation in corneal cell and animal models.

The limitations of this study include the relatively small number of isolates tested from each bacterial genus, the use of highly resistant but non-ocular *K. pneumoniae* isolates, and the use of non-ocular cell lines for cytotoxicity assessment, although these cells are recommended for standard toxicity testing. In addition, a ciprofloxacin uptake assay was not performed to determine whether Mel4 enhances the intracellular accumulation of ciprofloxacin in bacterial cells. Although the study primarily focused on ocular pathogens, the safety of the individual agents and their combinations could not be evaluated using corneal epithelial cells, which would have provided a more clinically relevant model for ocular application.

## 4. Materials and Methods

### 4.1. Antimicrobial Agents and Bacterial Isolates

Culture media were purchased from Becton Dickinson (Franklin Lakes, NJ, USA). Fluoroquinolones (ciprofloxacin, levofloxacin), carbapenem (meropenem), cephalosporin (ceftazidime), aminoglycosides (gentamicin, tobramycin), and polymyxins (polymyxin B, colistin) were purchased from Sigma-Aldrich (St. Louis, MO, USA). Mel4 (KNKRKRRRRRRGGRRRR) was synthesized using a conventional solid-phase peptide synthesis protocol [39] and obtained from the Auspep Peptide Company (Tullamarine, Victoria, Australia). The purity of the peptide was ≥90%. ESKAPEE isolates were obtained from the microbiology strain bank at the School of Optometry and Vision Science (Table 7) and selected based on their isolation from contact lens-related adverse events or known antibiotic resistance traits (in the case of *K. pneumoniae*).

### 4.2. MIC and MBC Determination

The antimicrobial activity of the tested compounds was evaluated using the broth microdilution method [27]. Briefly, strains were inoculated into trypticase soy broth (TSB) and incubated at 37 °C in a shaking incubator (120 rpm) for 18–24 h. Overnight bacterial cultures were washed with PBS and adjusted to approximately 1 × 10^8^ CFU/mL, then diluted to a final inoculum of 5 × 10^5^ CFU/mL in cation-adjusted Mueller–Hinton broth. Two-fold serial dilutions of each antimicrobial were prepared in 96-well plates and incubated at 37 °C for 18–24 h [45]. Minimum bactericidal concentrations (MBCs) were determined by subculturing onto trypticase soy agar plates, followed by incubation at 37 °C for 18–24 h. The MBC was defined as the lowest concentration producing a ≥99.9% reduction in viable cells [46]. All experiments were performed in triplicate.

### 4.3. Checkerboard Assay for Synergy Testing

Synergy between membrane-active antimicrobials and conventional antibiotics was assessed using checkerboard assays [44,47]. Colistin or Mel4 was combined with ciprofloxacin, gentamicin, tobramycin, ceftazidime or meropenem against selected resistant ESKAPEE isolates. Interactions were interpreted using the fractional inhibitory concentration index (FICI), calculated as FIC drug A + FIC drug B, where FIC = MIC in combination/MIC alone. FICI values were interpreted as synergy (≤0.5), indifference (>0.5–4) or antagonism (>4). Assays were repeated in at least two independent experiments [48].

### 4.4. Time–Kill Assay

Time–kill assays were performed to confirm selected synergistic combinations [47]. Bacterial suspensions were added to cation-adjusted Mueller–Hinton broth containing individual antimicrobials or combinations at sub-inhibitory concentrations to achieve an initial inoculum of 1 × 10^6^ CFU/mL in a final volume of 1ml. Cultures were incubated at 37 °C with a shaking incubator, and about 100 µL of samples were collected at 0, 3, 6, 9, 12, and 24 h. About 100-fold serial dilutions were done, and about 20 µL of aliquot was plated on Mueller–Hinton agar, and viable counts were determined at each time point to characterize the temporal antibacterial effects of each agent and the corresponding combination. However, overall bactericidal activity was determined and defined as a ≥3 log_10_ CFU/mL reduction from the initial inoculum, and synergy as a ≥2 log_10_ CFU/mL reduction compared with the most active single agent at 24 h [49].

### 4.5. Biofilm Disruption Assay and Confocal Microscopy

Biofilm disruption was assessed using preformed *P. aeruginosa* biofilms. Bacterial suspensions were added to 96-well plates and incubated statically at 37 °C for 24 h to allow biofilm formation. Planktonic cells were removed, and biofilms were treated with ciprofloxacin, Mel4 or their combinations at concentrations ranging from 0.25× to 2× MIC for a further 24 h. Biofilm biomass was quantified using crystal violet staining. For confocal laser scanning microscopy, treated biofilms were stained with the Live/Dead BacLight bacterial viability kit and imaged using a Nikon AXR confocal microscope. Images were analyzed using Fiji ImageJ software (1.54r) [50].

### 4.6. Cytotoxicity and Hemolysis Assays

Cytotoxicity was assessed using L929 fibroblast cells exposed to individual antimicrobials or combinations for 24 h [51]. Cell viability was measured using the MTT assay at 570 nm and expressed relative to untreated controls. Hemolytic activity was evaluated using horse red blood cells exposed to ciprofloxacin, Mel4, or their combinations [20]. Horse red blood cells (RBCs) were used as the model system Lu et al., 2022 [52]; Willcox et al., 2008 [53]; Yasir et al., 2019 [51]. PBS-treated cells served as the negative control, and Milli-Q water-treated cells as the positive control.

### 4.7. Cytoplasmic Membrane Permeability Assay

The method was adopted from Kuppusamy, R et al. [54] with slight modifications. Bacterial cytoplasmic membrane permeability was determined using the membrane potential-sensitive dye diSC3-5 (3,3′-dipropylthiadicarbocyanine iodide), which penetrates bacterial cells depending on the membrane potential gradient of the cytoplasmic membrane. Bacteria were grown in MHB to mid-log phase by incubating with shaking at 37 °C for 18–24 h. Following incubation, bacteria were washed with 5 mM HEPES containing 20 mM glucose, pH 7.2, and resuspended in the same buffer to an OD_600_ of 0.05–0.06, which gave 1 × 10^7^ CFU/mL. The dye diSC3-5 was added at 2 µM to the bacterial suspension. The suspensions were incubated at room temperature for 1 h in the dark for maximum dye uptake by the bacterial cells. Then, 100 mM KCl was added to balance the K+ outside and inside the bacterial cell to prevent further uptake or outflow of the dye. For Gram-negative bacteria, 0.5 mM EDTA was used to destabilize the lipopolysaccharides-Mg^2+^-Ca^2+^ complex to help in dye penetration without affecting bacterial growth. A total of 100 µL of bacterial suspension was added to a 96-well microtiter plate with an equal volume of antimicrobial compounds. DMSO (20%) was set as a positive control, while dye and only bacterial cells were set as a negative control. Fluorescence was measured with a luminescence spectrophotometer at 2 min intervals at an excitation wavelength of 622.

## 5. Conclusions

This study evaluated the antimicrobial efficacy of traditional antibiotics and membrane-active peptides (colistin, peptide Mel4) individually and in combination against both planktonic and biofilm-associated ESKAPEE pathogens. While many clinical isolates, particularly *P. aeruginosa*, *A. baumannii*, and *K. pneumoniae*, exhibited high-level resistance to conventional antibiotics, combination therapy with Mel4 or colistin significantly enhanced antimicrobial activity. Notably, synergistic interactions were observed between Mel4 or colistin and ciprofloxacin, meropenem, or at sub-inhibitory concentrations, resulting in marked reductions in MIC values and improved antibiofilm effects. Importantly, these combinations maintained high selectivity toward mammalian cells, with minimal cytotoxicity and hemolysis, even though these are not definitive results. The complementary mechanisms of Mel4 facilitating antibiotic entry and disruption of bacterial defense systems need further investigation. Overall, this study highlights the potential of polymyxin-based (and perhaps in the future Mel4-based) combination therapies as a promising strategy to combat multidrug-resistant ESKAPEE pathogens, offering both potent antibacterial and antibiofilm activity while minimizing toxicity to host cells. These findings support further investigation into the clinical applicability of antimicrobial peptide–antibiotic combinations as an alternative approach to overcoming multidrug resistance.

## Figures and Tables

**Table 1:** Minimum inhibitory (MIC) and minimum bactericidal (MBC) concentrations of conventional antibiotics against Gram-negative ESKAPEE isolates.

Isolates	Strains	Fluoroquinolones		Aminoglycosides		Cephalosporin	Carbapenem
		Ciprofloxacin	Levofloxacin	Gentamicin	Tobramycin	Ceftazidime	Meropenem
		MIC/MBC (mg/L)					
*P. aeruginosa*	27853	0.5(S)/0.5	0.5(S)/0.5	0.25(S)/0.5	0.25(S)/0.5	1(S)/1	0.25(S)/0.5
	PAO1	0.5(S)/0.5	0.5(S)/0.5	0.5(S)/1	0.5(S)/0.5	1(S)/1	0.25(S)/0.5
	31	**16(R)/32**	**64(R)/128**	**>2048(R)/>2048**	**512(R)/512**	**32(R)/64**	4(I)/8
	33	**16(R)/32**	**64(R)/64**	**>2048(R)/>2048**	**512(R)/512**	**16(R)/32**	**32(R)/32**
	34	0.5(S)/0.5	2(I)/2	1(S)/1	**256(R)/256**	**32(R)/32**	**32(R)/64**
	123	0.25(S)/0.25	1(S)/1	8(I)/8	**8(R)/16**	2(S)/2	0.25(S)/0.25
	126	0.25(S)/0.25	1(S)/1	**>512(R)/>512**	**>512(R)/>512**	4(S)/4	0.5(S)/0.5
	221	**128(R)/256**	**64(R)/64**	**>1024(R)/>1024**	**>512(R)/>512**	**16(R)/64**	4(I)/8
	1270	**64(R)/128**	-	**>512(R)/>512**	**>512(R)/>512**	**512(R)/1024**	**16(R)/32**
*A. baumannii*	04	**64(R)/64**	**8(R)/8**	**512(R)/1024**	0.25(S)/0.5	**64(R)/128**	**16(R)/16**
	05	**64(R)/64**	**8(R)/8**	**1024(R)/1024**	0.25(S)/0.5	**128(R)/256**	**64(R)/64**
	06	**128(R)/128**	**16(R)/16**	**1024(R)/1024**	0.25(S)/0.5	**>1024(R)/>1024**	**64(R)/64**
	07	**32(R)/64**	4(I)/8	**128(R)/256**	0.5(S)/0.5	**128(R)/256**	**128(R)/128**
	08	**64(R)/64**	4(I)/8	**>1024(R)/>1024**	**>1024(R)/>1024**	**128(R)/128**	**512(R)/512**
*K. pneumoniae*	JIE2709	**64(R)/128**	**>512(R)/>512**	**>1024(R)/>1024**	**16(R)/32**	**512(R)/512**	**>512(R)/>512**
	JIE2713	**128(R)/128**	**16(R)/16**	**512(R)/512**	**>512(R)/>512**	**>2048(R)/>2048**	**512(R)/512**
	JIE4212	**64(R)/64**	**8(R)/8**	**128(R)/128**	**32(R)/64**	**1024(R)/2024**	**8(R)/32**
*E. coli*	10	0.06(S)/0.06	0.06(S)/0.06	0.5(S)/0.5	0.5(S)/0.5	2(S)/2	0.06(S)/0.06
	11	0.06(S)/0.06	0.25(S)/0.25	0.5(S)/0.5	0.5(S)/0.5	1(S)/1	0.06(S)/0.06
	15	0.06(S)/0.06	0.125(S)/0.125	1(S)/2	0.5(S)/0.5	1(S)/2	0.06(S)/0.06
*E. cloacae*	02	0.06(S)/0.06	0.06(S)/0.06	0.5(S)/0.5	0.5(S)/1	8(I)/8	0.5(S)/0.5
	05	0.06(S)/0.06	0.125(S)/0.125	0.5(S)/0.5	0.5(S)/1	8(I)/8	0.06(S)/0.06

R = resistant, S = susceptible, I = intermediate according to CLSI 2020 guidelines [27]. Bold font indicates resistance to the antibiotic. - = not tested.

**Table 2:** MIC and MBC values of membrane-active antimicrobials against Gram-negative isolates.

Isolate	Strain	Polymyxins		AMP
		Polymyxin B	Colistin	Mel4
		MIC/MBC (mg/L)		
*P. aeruginosa*	31	2(I)/2	2(I)/2	62.5/125
	33	2(I)/2	2(I)/2	62.5/125
	34	2(I)/4	1(I)/2	62.5/125
	123	**4(R)/4**	2(I)/2	3.9/7.8
	126	2(I)/2	1(I)/1	250/250
	221	2/(I)/2	2/(I)/2	125/250
	1270	-	1(I)/1	>250/>250
*A. baumannii*	04	0.5(I)/0.5	2(I)/2	125/250
	05	0.5(I)/0.5	1(I)/1	250/500
	06	1(I)/1	1(I)/1	125/250
	07	1(I)/1	1(I)/1	125/250
	08	0.5(I)/0.5	1(I)/2	250/250
*K. pneumoniae*	JIE2709	1(I)/1	1(I)/1	62.5/250
	JIE2713	1(I)/2	1(I)/1	31.25/62.5
	JIE4212	1(I)/2	1(I)/1	125/125
*E. coli*	10	0.5(S)/1	1 (I)/1	250/250
	11	0.5(S)/1	1(I)/1	>500/>500
	15	2(S)/2	2(I)/2	125/250
*E. cloacae*	02	**32(R)/32**	**32(R)/32**	250/250
	05	**32(R)/32**	**32(R)/32**	125/250

R = resistant, S = susceptible, I = intermediate according to CLSI 2020 guidelines [27]. Bold font indicates resistance to the antibiotic. - = not tested.

**Table 3:** Effect of combinations of aminoglycoside and cephalosporin against Gram-negative pathogens.

Bacteria	Isolate	Combinations Drug A + Drug B	MIC (mg/L)				FIC			Drug Interaction
			Drug A	Drug A + Drug B	Drug B	Drug B + Drug A	Drug A	Drug B	FICI	
*P. aeruginosa*	31	Gentamicin + Ceftazidime	4096	512	32	8	0.125	0.25	0.38	Synergy
	33	Gentamicin + Ceftazidime	4096	512	16	8	0.125	0.5	0.63	Additive
	34	Tobramycin + Ceftazidime	256	16	8	2	0.06	0.25	0.31	Synergy
	123	Gentamicin + Ceftazidime	8	2	2	1	0.25	0.5	0.75	Additive
*A. baumannii*	04	Gentamicin + Ceftazidime	512	128	64	16	0.25	0.25	0.5	Synergy
	08	Gentamicin + Ceftazidime	4096	8	128	64	0.002	0.5	0.5	Synergy
*K. pneumoniae*	JIE2709	Tobramycin + Ceftazidime	16	8	512	16	0.5	0.03	0.5	Synergy
	JIE4212	Gentamicin + Ceftazidime	256	32	1024	512	0.125	0.5	0.63	Additive

**Table 4:** MIC values and Synergistic effects of antibiotic–colistin combinations against drug-resistant Gram-negative pathogens.

Isolate	Combinations Drug A + Drug B	MIC (mg/L)				FIC			
		Drug A	Drug A + Drug B	Drug B	Drug B + Drug A	Drug A	Drug B	FICI	Drug Interaction
PA31	Ciprofloxacin + Colistin	16	1	2	2	0.06	1	1.06	Indifference
	Gentamicin + Colistin	4096	1	2	2	0.0002	1	1.000	Indifference
	Tobramycin + Colistin	512	1	2	2	0.002	1	1.002	Indifference
	Ceftazidime + Colistin	32	0.125	2	1	0.004	0.5	0.51	Synergy
	Meropenem + Colistin	512	256	2	1	0.5	0.5	1	Indifference
PA33	Ciprofloxacin + Colistin	16	1	2	1	0.5	0.25	0.75	Additive
	Gentamicin + Colistin	4096	1	2	2	0.0002	1	1.0002	Indifference
	Tobramycin + Colistin	512	512	2	0.008	1	0.02	1.02	Indifference
	Ceftazidime + Colistin	16	4	2	1	0.25	0.5	0.75	Additive
	Meropenem + Colistin	32	16	2	0.125	0.5	0.0625	0.56	Additive
PA 34	Tobramycin + Colistin	256	128	1	0.5	0.5	0.5	1	Indifference
	Ceftazidime + Colistin	32	8	2	0.25	0.25	0.25	0.5	Synergy
	Meropenem + Colistin	32	8	2	0.25	0.25	0.25	0.5	Synergy
PA 123	Gentamicin + Colistin	8	1	2	0.25	0.125	0.063	0.19	Synergy
PA 221	Ciprofloxacin + Colistin	128	0.5	2	2	0.004	1	1.004	Indifference
	Gentamicin + Colistin	4096	1	2	2	0.0002	1	1.000	Indifference
	Ceftazidime + Colistin	16	4	2	0.25	0.25	0.125	0.375	Synergy
PA1270	Ciprofloxacin + Colistin	64	0.5	1	1	0.01	1	1.01	Indifference
	Gentamicin + Colistin	2048	0.5	1	1	0.0002	1	1.000	Indifference
	Tobramycin + Colistin	2048	2	1	0.5	0.001	0.5	0.501	Synergy
	Ceftazidime + Colistin	512	0.5	1	1	0.001	1	1.001	Indifference
	Meropenem + Colistin	16	0.5	1	1	0.03	1	1.03	Indifference
AB 04	Ciprofloxacin + Colistin	64	0.5	2	1	0.01	0.5	0.51	Synergy
	Gentamicin + Colistin	512	64	2	0.5	0.125	0.25	0.375	Synergy
	Ceftazidime + Colistin	64	2	2	1	0.031	0.5	0.531	Synergy
	Meropenem + Colistin	16	0.03	2	2	0.002	1	1.002	Indifference
AB 05	Gentamicin + Colistin	1024	256	1	0.25	0.25	0.25	0.5	Synergy
	Ceftazidime + Colistin	128	32	1	0.5	0.25	0.5	0.75	Additive
	Meropenem + Colistin	64	0.125	1	1	0.002	1	1.002	Indifference
AB06	Ciprofloxacin + Colistin	128	0.125	1	1	0.001	1	1.001	Indifference
	Gentamicin + Colistin	1024	128	1	0.25	0.125	0.25	0.375	Synergy
	Ceftazidime + Colistin	4096	256	1	0.5	0.625	0.5	0.563	Synergy
	Meropenem + Colistin	64	16	1	0.125	0.25	0.125	0.375	Synergy
AB07	Gentamicin + Colistin	128	8	1	0.5	0.0625	0.5	0.563	Synergy
	Ceftazidime + Colistin	128	64	1	0.125	0.5	0.125	0.625	Additive
AB08	Ciprofloxacin + Colistin	64	8	1	0.5	0.125	0.5	0.625	Additive
	Gentamicin + Colistin	4096	16	1	0.5	0.004	0.5	0.504	Synergy
	Tobramycin + Colistin	4096	128	1	0.5	0.03	0.5	0.53	Synergy
	Ceftazidime + Colistin	128	8	1	0.5	0.0625	0.5	0.563	Additive
	Meropenem + Colistin	64	0.03	1	0.5	0.0004	0.5	0.500	Synergy
KP JIE2709	Ciprofloxacin + Colistin	128	2	2	1	0.02	0.5	0.52	Synergy
	Ceftazidime + Colistin	512	2	2	0.25	0.002	0.125	0.125	Synergy
	Meropenem + Colistin	2048	2	2	0.25	0.001	0.125	0.126	Synergy
KP JIE2713	Ciprofloxacin + Colistin	128	8	1	1	0.06	1	1.06	Indifference
	Gentamicin + Colistin	4096	16	1	0.5	0.004	0.5	0.503	Synergy
	Tobramycin + Colistin	4096	8	1	0.5	0.002	0.5	0.51	Synergy
	Ceftazidime + Colistin	2048	1	1	0.5	0.0005	0.5	0.5005	Synergy
	Meropenem + Colistin	512	2	1	1	0.004	1	1.04	Indifference
KP JIE4212	Ciprofloxacin + Colistin	64	8	1	1	0.125	1	1.125	Indifference
	Gentamicin + Colistin	256	1	1	0.5	0.004	0.5	0.51	Synergy
	Tobramycin + Colistin	32	1	1	1	0.03	1	1.03	Indifference
	Ceftazidime + Colistin	2048	0.5	1	1	0.0002	1	1.000	Indifference
	Meropenem + Colistin	8	0.5	1	1	0.0625	1	1.0625	Indifference

PA = *Pseudomonas aeruginosa*, KP = *Klebsiella pneumoniae*, AB = *Acinetobacter baumanii*.

**Table 5:** MIC values and synergistic effects of standard antibiotics in combination with Mel4 against drug-resistant ESKAPEE pathogens.

Isolate	Combination Drug A + Drug B	MIC (mg/L)				FIC			
		Drug A	Drug A + Drug B	Drug B	Drug B + Drug A	Drug A	Drug B	FICI	Drug Interaction
PA31	Ciprofloxacin + Mel4	16	4	62.5	15.6	0.25	0.25	0.5	Synergy
	Tobramycin + Mel4	256	1	62.5	31.3	0.004	0.5	0.51	Synergy
	Ceftazidime + Mel4	32	16	62.5	31.3	0.5	0.5	1	Indifference
PA33	Ciprofloxacin + Mel4	16	4	62.5	15.6	0.25	0.25	0.5	Synergy
	Tobramycin + Mel4	512	0.2	62.5	62.5	0.0003	1	1.003	Indifference
	Ceftazidime + Mel4	16	1	62.5	31.3	0.06	0.5	0.56	Additive
PA34	Tobramycin+ Mel4	256	1	250	125	0.004	0.5	0.504	Synergy
	Meropenem + Mel4	32	0.25	250	125	0.01	0.5	0.501	Synergy
PA123	Gentamicin + Mel4	8	2	125	62.5	0.25	0.5	0.75	Additive
PA221	Ciprofloxacin + Mel4	128	32	125	15.6	0.25	0.125	0.375	Synergy
	Gentamicin + Mel4	4096	4	125	125	0.001	1	1.001	Indifference
PA1270	Ciprofloxacin + Mel4	64	2	1000	250	0.03	0.25	0.28	Synergy
	Meropenem + MEL4	16	2	1000	250	0.125	0.25	0.375	Synergy
AB 04	Ciprofloxacin + Mel4	64	0.5	125	62.5	0.008	0.5	0.508	Synergy
	Gentamicin + Mel4	512	1	125	62.5	0.002	0.5	0.502	Synergy
	Ceftazidime + Mel4	64	2	125	31.25	0.031	0.25	0.28	Synergy
	Meropenem + Mel4	16	4	125	62.5	0.25	0.5	0.75	Additive
AB05	Ciprofloxacin + Mel4	64	0.5	250	250	0.008	1	1.01	Indifference
	Ceftazidime + Mel4	128	32	250	15.6	0.25	0.06	0.31	Synergy
	Meropenem + Mel4	64	0.125	250	125	0.001	0.5	0.501	Synergy
AB06	Ciprofloxacin + Mel4	128	0.5	125	125	0.004	1	1	Indifference
	Gentamicin + Mel4	1024	1	125	125	0.001	1	1	Indifference
	Ceftazidime + Mel4	4096	128	125	31.3	0.031	0.25	0.28	Synergy
	Meropenem + Mel4	64	16	125	62.5	0.25	0.5	0.75	Additive
AB07	Ciprofloxacin + Mel4	32	0.5	125	125	0.02	1	1.02	Indifference
	Gentamicin + Mel4	128	1	125	125	0.008	1	1	Indifference
	Ceftazidime + Mel4	128	32	125	7.8	0.25	0.0625	0.313	Synergy
	Meropenem + Mel4	128	32	125	15.6	0.125	0.25	0.375	Synergy
AB08	Ciprofloxacin + Mel4	64	0.5	125	125	0.002	1	1.02	Indifference
	Gentamicin + Mel4	4096	1	250	125	0.0002	0.5	0.500	Synergy
	Ceftazidime + Mel4	128	32	250	62.5	0.25	0.25	0.5	Synergy
	Meropenem + Mel4	512	64	250	62.5	0.125	0.25	0.375	Synergy
KP2709	Ciprofloxacin + Mel4	128	64	62.5	31.3	0.5	0.5	1	Indifference
	Ceftazidime + Mel4	512	128	62.5	15.6	0.25	0.25	0.5	Synergy
KP2713	Ciprofloxacin + Mel4	128	32	62.5	15.6	0.25	0.25	0.5	Synergy
	Meropenem + Mel4	128	64	31.25	15.6	0.5	0.5	1	Indifference
KP4212	Ciprofloxacin + Mel4	64	32	125	62.5	0.5	0.5	1	Indifference
	Gentamicin + Mel4	256	64	125	62.5	0.25	0.5	0.75	Additive
	Meropenem + Mel4	64	16	125	62.5	0.25	0.5	0.75	Additive
EC002	Levofloxacin + Mel4	0.06	0.06	250	0.98	1	0.003	1.003	Indifference
	Ceftazidime + Mel4	8	4	125	1	0.5	0.01	0.51	Synergy

PA = *Pseudomonas aeruginosa*, KP = *Klebsiella pneumoniae*, AB = *Acinetobacter baumanii*, EC = *Enterobacter cloacae*.

**Table 6:** Horse red blood cell hemolysis of ciprofloxacin, Mel4, and their combinations across concentration gradients.

Mel4 (mg/L)	Lysis (%)	CIP (mg/L)	Lysis (%)	CIP + Mel4 (mg/L)	Lysis (%)
512	0	512	39	48 + 187 (3× MIC)	1
256	0	256	7	32 + 125 (2× MIC)	1
128	0	128	0	16 + 62.5 (1× MIC)	0
64	0	64	0	8 + 31.25 (1/2× MIC)	0
32	0	32	0	4 + 15.6 (1/4× MIC)	0

FIC combination for *P. aeruginosa* 31 and 33.

**Table 7:** Selected Gram-negative pathogens used for this antimicrobial sensitivity testing.

Bacteria	Strain	Resistance Genes	Isolation Source	Reference
*P. aeruginosa*	31	*aph(6)-Id*, *aph(3′)-Ib*, *aph(3′)-llb*, *crpP*, *blaPAO*, *qacEdelta1*, *sul1*, *tet(G)*, *catB7*, *fosA*	Corneal infection	[40]
	33	*aph(6)-Id*, *aph(3′)-Ib*, *crpP*, *blaPAO*, *blaOXA-50*, *qacEdelta1*, *sul1*, *tet(G)*, *catB7*, *fosA*	Corneal infection	[41]
	34	*aac(3)-IId*, *aph(3′)-IIb*, *blaOXA-488*, *blaPAO*, *catB7*, *fosA*, *PDC-39*, *arnA*, *arnT*, *basR*, *basS*, *cprR*, *cprS*, *crpP*, *parC*, *soxR*, *vanW*	Corneal infection	[40,42]
	123	*aph(3′)-IIb*, *blaPAO*, *blaOXA-486*, *blaOXA-903*, *blaOXA-1028*, *catB7*, *fosA*, *PDC-374*, *arnA*, *arnT*, *cprR*, *basR*, *basS*, *cprS*, *parC*, *soxR*, *vanW*	Corneal infection	[40,42]
	126	*aph(3′)-IIb*, *blaOXA-50*, *blaOXA-396*, *blaOXA-494*, *blaOXA-850*, *blaPAO*, *catB7*, *fosA*, *PDC-3*, *arnA*, *arnT*, *basR*, *basS*, *cprR*, *cprS*, *parC*, *soxR*, *vanW*	Corneal infection	[40,42]
	221	*aac [3]-IId*, *aac(6′)-Ib9*, *aadA*, *aadA1/10*, *aadA11*, *aph (3′)-IIb*, *aph(3″)-Ib*, *aph [6]-Id/IId*, *blaOXA-395*, *blaPAO*, *catB7*, *fosA*, *LCR-1*, *PDC-36*, *arnA*, *arnT*, *basS*, *cprR*, *cprS*, *crpP*, *gyrA*, *parC*, *qnrVC1*, *rmtD*, *rmtD2*, *soxR*, *sul1*, *tet(G)*, *vanW*	Corneal infection	[40,42,43]
	1270	*bla*, *blaP*, *fimD*, *higB-2*, *intA_1–4*, *intS_2*, *mdtA_4*, *pilY1*, *tetA_1*, *tetR*, *traG_2*, *xcpT_2*, *ydhC_1–3*, *ydhP_1*	Corneal infection	[42]
*A.baumannii*	04	NK	Contact lens of an asymptomatic wearer	This study
	05	NK	Contact lens during corneal inflammation	[44]
	06	NK	Contact lens during corneal inflammation	[44]
	07	NK	Corneal infection	[44]
	08	NK	Unknown	[44]
*E. coli*	10	NK	Contact lens during corneal inflammation	[44]
	11	NK	Contact lens during corneal inflammation	[44]
	15	NK	Unknown	[44]
*E. cloacae*	02	NK	Contact lens of an asymptomatic wearer	This study
	05	NK	Contact lens of an asymptomatic wearer	[44]
*K. pneumoniae*	JIE2709	*blaSHV*, *blaTEM*, *blaKPC*, *aac(6)-Ib*, *aphA1*, *dfrA12*, *aadA2*, *sul1*	Urine	[44]
	JIE2713	*aac(6)*, *blaCTX-M-15*, *blaNDM-1*, *blaOXA-30*	Unknown	[44]
	JIE4212	*ampH*, *blaTEM-30*, *blaOXA-1*, *blaOXA-9*, *blaSHV-28*	Clinical isolate (site not reported)	[43,44]

NK, not known; blaTEM, Temoniera; blaOXA, oxacillinase; blaSHV, sulfhydryl; blaCTX-M, cefotaximase-munich β-lactamase; aac, aminoglycoside acetyltransferase; blaNDM, New Delhi metallo-β-lactamase; dfr, dihydrofolate reductase; blaKPC, *K. pneumoniae* carbapenemases; aph, aminoglycoside phosphotransferase; aad, aminoglycoside adenyltransferase; sul, sulfonamide-resistant dihydropteroate synthase; catB, chloramphenicol acetyltransferaseB; qacEdelta, quarternary ammonium compound efflux gene delta; fosA, fosfomycin thiol transferase; rmt, ribosomal methyltransferase; arnA, 4-amino-4-deoxy-L-arabinose biosynthesis protein ArnA; arnT, L-Ara4N transferase ArnT; basR, response regulator BasR; basS, sensor kinase BasS; cprR, two-component response regulator CprR; cprS, two-component sensor kinase CprS; soxR, superoxide response transcriptional regulator SoxR; vanW, vancomycin resistance-associated protein W; tet, tetracycline efflux pump; PDC, *Pseudomonas*- derived cephalosporinase; blaPAO, *Pseudomonas aeruginosa* cephalosporinase; gyrA, DNA gyrase; parC, topoisomerase IV subunit A; qnr, quinolone resistance protein; crpP, ciprofloxacin resistance protein; vanW, vancomycin resistance-associated protein W.

## Data Availability

All data analyzed during this study were included in this article. Data that support the findings of this study are also available from the corresponding author upon reasonable request.

## Abbreviations

WHO: World Health Organization, MDR: Multidrug Resistance, XDR: Extensively drug-resistant, ESKAPEE: Enterococcus faecium, Staphylococcus aureus, Klebsiella pneumoniae, Acinetobacter baumannii, Pseudomonas aeruginosa, Enterobacter spp., and Escherichia coli, CDC: Centers for Disease Control and Prevention, FICI: Fractional Inhibitory Concentration Index, CAMHB: Cation-Adjusted Mueller-Hinton Agar, DMEM: Dulbecco’s Modified Eagle Medium, CLSI: Clinical Laboratory Standards Institute. MTT: 3-(4,5-dimethylthiazol-2-yl)-2,5-diphenyltetrazolium bromide., DMSO: Dimethyl sulfoxide.
